# Contemporary Management of Malignancies of the Parotid Gland: A State‐of‐the‐Art Review

**DOI:** 10.1002/ohn.70170

**Published:** 2026-02-16

**Authors:** Anthony Tang, Vanessa Helou, Shaum S. Sridharan, Renata Ferrarotto, Jessica L. Geiger, Dan P. Zandberg, Diana Bell, Heath Skinner, Matthew E. Spector, Eugene N. Myers, Kevin J. Contrera

**Affiliations:** ^1^ University of Pittsburgh School of Medicine Pittsburgh Pennsylvania USA; ^2^ Department of Otolaryngology University of Pittsburgh School of Medicine Pittsburgh Pennsylvania USA; ^3^ Department of Thoracic/Head and Neck Medical Oncology The University of Texas MD Anderson Cancer Center Houston Texas USA; ^4^ Department of Medical Oncology Cleveland Clinic Cleveland Ohio USA; ^5^ Department of Medical Oncology University of Pittsburgh School of Medicine Pittsburgh Pennsylvania USA; ^6^ Department of Pathology University of Pittsburgh School of Medicine Pittsburgh Pennsylvania USA; ^7^ Department of Radiation Oncology University of Pittsburgh School of Medicine Pittsburgh Pennsylvania USA

**Keywords:** cancer, immunotherapy, parotid, salivary gland, targeted therapy

## Abstract

**Objectives:**

To provide a systematic update on the contemporary management of malignancies of the parotid gland (MPG), focusing on evidence‐based practices and emerging therapeutics.

**Data Sources:**

PubMed literature search.

**Review Methods:**

A search protocol was designed according to the Preferred Reporting Items for Systematic Reviews and Meta‐analyses (PRISMA) process for articles published between August 1, 2019, and August 1, 2024. Primary exclusion criteria were non‐English text, abstracts, and case reports/series. Secondary inclusion criteria were articles specific to MPG diagnosis, histology, outcomes, and management, including surgery, radiotherapy, chemotherapy, immunotherapy, and targeted therapy.

**Conclusion:**

A total of 1614 articles were identified, and 137 articles were included in the final review. Most studies on low‐grade MPG found that surgery alone was noninferior to surgery and adjuvant radiotherapy for local control. Elective neck dissection provided survival benefits for high‐grade, but not low‐grade, MPG. Most MPG are chemotherapy‐resistant. Adjuvant chemotherapy provides limited survival benefits while significantly worsening quality of life and, thus, should be used only in select patients. Immunotherapy and targeted therapies for markers, including HER‐2, TRK, and androgen receptors, have shown promising results in the treatment of advanced MPG.

**Implications for Practice:**

Advances in the treatment of MPG have improved survival while minimizing treatment toxicity and improving quality of life. Future studies are needed to emphasize personalized oncologic treatment for patients with these rare malignancies.

Malignancies of the parotid gland (MPG) are rare, accounting for 6% to 8% of all head and neck cancer (HNC).^1^ An estimated 60% to 75% of salivary gland cancers occur in the parotid gland. MPG are clinically diverse and are comprised of a heterogeneous group of tumors. Their varied histopathology and presentation often make diagnosis and treatment challenging.

In recent years, new investigations have provided evidence for changes in surgical resection goals surrounding MPGs, as well as the management of regional metastasis and the effectiveness of adjuvant treatments. Studies have also begun to explore the utility of novel therapies, including immunotherapy and targeted therapies. Given the critical advancements that have been developed, otolaryngologists need to be familiar with the contemporary management of MPG. This state‐of‐the‐art review summarizes the management of MPG and provides a foundation for how otolaryngologists can continue to improve outcomes for these patients.

## Methods

A search protocol was designed according to the Preferred Reporting Items for Systematic Reviews and Meta‐Analyses (PRISMA) process.^2^ First, the concepts relevant to the search topic were identified by two investigators (AT and KJC) as “(parotid[title] OR parotid gland[title] OR salivary gland[title]) AND (malignancy OR malignant OR cancer OR carcinoma) AND (treatment OR management OR therapy).” These concepts were used to identify medical subject heading (MeSH) terms and keywords in PubMed. Identified publications then underwent independent review by 1 investigator (AT), who conducted title and abstract reviews to screen articles for exclusion. Two independent reviewers participated in the full‐text review (AT and VH) with a third serving as the tiebreaker for disagreements (KJC). Exclusion criteria included articles published more than 5 years ago, non‐English text, abstracts, and articles not specific to MPG diagnosis, histology, surgical management, radiation therapy, chemotherapy, immunotherapy, or targeted therapy.

**Figure 1:**
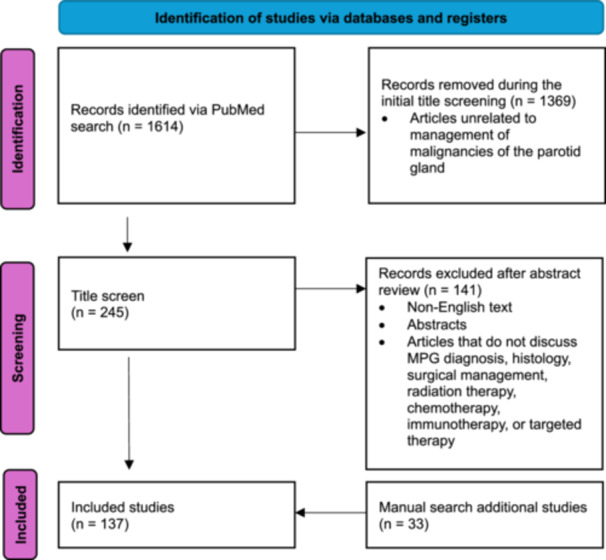
PRISMA. MPG, malignancies of parotid gland.

Relevant citations were identified, and MeSH terms and keywords were extracted for inclusion in the next search iteration until all applicable search terms were captured. PubMed and Medline databases were searched on August 25, 2024. A total of 1614 articles were identified. After the title review, 245 articles were further evaluated. Abstracts were reviewed for relevance to our inclusion criteria. 141 were deemed irrelevant and removed, leaving 104 articles. An additional 33 articles were found via manual search for a total of 137 studies (see Figure 1 for details).

## Discussion

### Diagnosis

#### Free Needle Aspiration (FNA)

The initial diagnostic tool for evaluating a mass in the parotid gland is an FNA biopsy, often guided by ultrasound. The American Society of Clinical Oncology (ASCO) guidelines for salivary gland malignancies strongly recommend performing tissue biopsy to support the distinction between malignant and nonmalignant salivary gland lesions.^3^ Moreover, the cytology of FNA samples and HPV status of cytology have emerged as a valuable diagnostic tool for patients with metastases to the parotid gland.^4^ However, FNA is often operator‐dependent, leading to diagnostic discrepancies. While FNA has high specificity for diagnosing MPG, its sensitivity is lower and more variable.^5^, ^6^, ^7^ Multiple studies have reported on the positive predictive value (PPV), negative predictive value (NPV), sensitivity, and specificity for FNA in the evaluation of a parotid mass as malignant or benign, the PPV ranged from 75% to 98.6%, the NPV ranged from 94.3% to 96.7%, the sensitivity ranged from 85.7% to 90%, and the specificity ranged from 93.7% to 99.5%. Specifically, for diagnosing mucoepidermoid carcinoma (MEC), Iftikhar et al found that over 50% of cases showed discordant results between cytology and histology.^8^, ^9^, ^10^, ^11^

To improve the consistency of FNA reporting, the Milan System for Reporting Salivary Gland Cytopathology (MSRSGC) was established in 2018 as a standardized, evidence‐based system.^12^, ^13^ MSRSGC showed high interobserver agreement.^14^ Moreover, it was found that applying the MSRSGC to cystic salivary gland lesions improved patient management by preventing unnecessary surgery for nonneoplastic conditions.^15^

FNA also has a high misdiagnosis rate of malignancy in patients with low‐ to intermediate‐grade MPG.^16^ Other diagnostic methods, including incisional biopsy and core needle biopsy, show similar accuracy for diagnosing MPG compared to FNA.^17^, ^18^ While FNA is a quick, minimally invasive, and relatively painless procedure commonly done in outpatient settings, some argue that excision is necessary regardless of test results.^19^ However, others suggest that FNA results can influence preoperative counseling, surgery timing, and the extent of surgery.^20^

#### Imaging and Other Diagnostic Tools

Doppler ultrasound is another diagnostic method for MPG due to its high degree of accuracy in differentiating benign and malignant parotid tumors.^21^ Ultrasound alone has been shown to have a PPV of 64.7%, a NPV of 94.1%, a sensitivity of 81.8%, and a specificity of 52.6% for detecting malignancy.^22^ When swelling of the parotid gland persists for 3 weeks, ultrasound is recommended to confirm a tumor and exclude other diagnoses. In addition, the polar vessel sign on ultrasound, defined as a prominent blood vessel without branches penetrating the tumor, has been shown to have a high diagnostic specificity of 100% and sensitivity of 71.4% for diagnosing adenoid cystic carcinoma (ACC).^23^

Magnetic resonance imaging (MRI) with diffusion‐weighted and dynamic contrast‐enhanced techniques is recommended. MRI provides good tissue contrast resolution, helping to assess tumor characteristics, local invasion, nerve diffusion, and lymph node metastasis.^24^ A recent meta‐analysis showed that multimodal MRI has high specificity and sensitivity for diagnosing MPG, although with significant heterogeneity.^25^ Kuang et al expanded upon this, showing that MRI alone has a PPV of 75.8%, a NPV of 95%, a sensitivity of 95.5%, and a specificity of 71.9%.^22^ When combining ultrasound and MRI, this leads to a PPV of 84.6%, a NPV of 95%, a sensitivity of 72.7%, and a specificity of 94.8%.

In the case of histologically proven malignancy or a highly suspicious lesion, a computed tomography (CT) scan of the neck and chest is also recommended to evaluate the tumor and search for metastases.^26^ Although fluorodeoxyglucose‐positron emission tomography (FDG‐PET) scans have been used, there is no evidence supporting routine use for initial diagnosis, extension assessment, treatment response evaluation, staging of recurrence, or follow‐up in salivary gland tumors.^3^, ^26^ However, hybrid PET can be useful in preoperative assessment, as a high standardized uptake value of the primary tumor may guide clinical decisions, particularly for MPG with high‐grade histology and lymph node metastases.^27^ For ACC, prostate‐specific membrane antigen (PSMA)‐targeted PET is promising for detecting lesions and may be suitable for targeted radioligand therapy.^28^

### Histology‐Specific Updates

#### MEC

MEC is the most common MPG. It predominantly affects White individuals and exhibits greater aggressiveness in adults than in the pediatric population.^1^ Even after surgical resection, the overall survival (OS) is poorer in adults than in children.^29^ Furthermore, MECs are classified into low, intermediate, and high‐grade categories, with generally worse outcomes for higher‐grade histologies. Nance et al found that intermediate‐grade MEC aligns more closely with low‐grade MEC in terms of survival, suggesting that treatment for intermediate‐grade tumors should be the same as that for low‐grade MEC.^30^

Multiple studies have found correlations between survival and histologic grade, nodal status, distant metastasis, intraparotid metastasis, perineural invasion (PNI), and age.^31^, ^32^, ^33^ These prognostic factors can help guide the most appropriate treatment and surveillance. One study suggested PNI should qualify as high‐grade in revised staging.^33^ Distant metastasis remains the most common form of recurrence in MEC, with high‐grade histology, lymphovascular invasion, and PNI recognized as significant risk factors for distant recurrence.^34^ CRTC1‐MAML2 and CRTC1/3‐MAML2 fusion genes have emerged as promising biomarkers for predicting favorable prognosis for patients with salivary gland MEC, with CRTC1‐MAML2 gene expression found to have a higher positive rate in low‐grade MEC.^35^, ^36^ HER2 overexpression correlates with poor prognosis in salivary MEC and has the potential to serve as a therapeutic target.^37^ Low‐ and intermediate‐grade MEC have significantly improved survival compared to high‐grade MEC. However, the rate of correct assessment of grade by FNA cytology is poor, reported to be 20% in one study.^38^ Taniuchi et al showed that in 12 patients with high‐grade MEC without invasion of the main trunk of the facial nerve, total sacrifice of the facial nerve led to a low local recurrence rate (17%), suggesting a potential recurrence benefit with facial nerve sacrifice.^38^

#### ACC

ACC of the parotid gland is a rare malignancy, representing approximately 1% of all cancers of the head and neck.^39^ Despite its overall slow growth, ACC is characterized by high infiltration and an exceptional propensity for PNI. Extensive perineural spread, reported in 29% to 62% of cases, is strongly associated with local recurrence as well as significant pain.^40^, ^41^ The propensity of ACC to spread to the skull base necessitates the inclusion of adjuvant radiation therapy in treatment plans.^42^ However, despite complete surgical resection and radiation therapy, long‐term survival remains guarded due to the high likelihood of late‐onset distant metastases, primarily to the lungs.^43^

Histologically, ACC exhibits diverse patterns, with the cribriform subtype being the most common and linked to a relatively favorable prognosis.^44^ In contrast, the solid subtype is associated with more aggressive disease and worse outcomes, even in early‐stage disease.^44^, ^45^ A meta‐analysis of 36 studies with 1608 patients by Li et al has highlighted the prognostic significance of P53 immunohistochemical (IHC) expression, associated with the solid subtype, with local recurrence, metastasis, and OS.^46^ In addition, Ferrarotto et al developed a two‐stage ACC classification system based on the IHC expression of P63 and MYC, where ACC‐I had strong upregulation of MYC and a worse prognosis and enrichment for mutations in NOTCH1‐4, SPEN, CREBPP, and EP300, and ACC‐II had upregulation of P63 and a less aggressive clinical course.^47^

Lymph node metastasis in ACC is relatively rare, with an overall incidence of approximately 7%. However, high‐grade and advanced‐stage cancers are associated with an increased risk of nodal involvement and worse survival outcomes.^48^, ^49^ A Surveillance, Epidemiology, and End Results (SEER) analysis by Ullah et al identified tumor grade, particularly grades III and IV, as the strongest predictors of poor prognosis.^50^

Molecular profiling has provided valuable insights into ACC's biology, revealing the challenges in identifying actionable genomic alterations. A recent retrospective study demonstrated that broader genomic profiling identified alterations in 87% of cases, with biomarker‐matched clinical trials available for 40%.^51^ This contrasts with smaller panels, which frequently fail to detect actionable alterations, emphasizing the importance of comprehensive genomic profiling.^51^ The t(6;9) translocation resulting in a MYB‐NFIB fusion oncogene is an example of a genetic alteration currently being investigated in clinical trial settings.^52^

#### Other Malignancies of the Parotid Gland

Salivary duct carcinoma (SDC) accounts for 4% to 5% of salivary gland malignancies and has a poor prognosis. Adenocarcinoma not otherwise specified (ANOS) accounts for 8% to 19% of salivary gland malignancies.^53^ However, Rooper et al found that 39% of malignancies that were initially diagnosed as NOS were reclassified as SDC using contemporary IHC profiling and diagnostic criteria by expert pathologists.^54^ SDC and ANOS are of particular importance due to their relatively high rate of HER2 and AR expression for targeted treatments.^53^ The majority of parotid squamous cell carcinomas (SCC) are metastases from cutaneous lesions of the face and scalp. Compared to primary cancers of the parotid gland, patients suffering from cutaneous SCC (CSCC) metastases to the parotid gland present with significantly higher age and worse survival.^55^ Overall, MPGs include a heterogeneous group of histological subtypes. However, the management of these diseases has a shared consideration of the importance of histological grade.

### Surgical Management

#### Surgical Goals

The goal of surgical management of MPG is negative margins while limiting morbidity. Studies have established that a positive margin status is associated with worse local control and survival. However, a recent study by Hanson et al looking at patients with malignancies of the salivary gland, found that margin status was not an independent factor associated with poorer outcomes, after controlling for tumor stage, histologic grade, and the use of adjuvant radiotherapy.^56^ In addition, patients with close margins with low‐ or intermediate‐grade histologic types with stage I/II cancers may be managed safely without the need for adjuvant radiotherapy.^56^ Zhu et al built a risk classification system using SEER data of 5077 patients with MPG that stratifies patients with MPG into three risk groups after surgery based on total points accounting for age, marital status, gender, tumor grade, histology type, TNM staging, surgery type, adjuvant radiotherapy, and adjuvant chemotherapy.^57^ In their validation cohort, their risk classification system performed better than TNM staging in predicting OS. Overall, studies stratifying risk in MPGs have consistently found multiple factors to be associated with a worse prognosis; these factors include tumor grade, tumor size, tumor histology, perineural and angiolymphatic invasion, and nodal or metastatic disease at presentation.

Multiple studies have shown that for high‐grade MPG, total parotidectomy should be completed, and that adjuvant therapy is recommended only in patients with specific risk factors.^58^, ^59^ Ketterer et al looked at 102 MPG and recommended complete parotidectomy for high‐grade and stage T3‐T4 low‐grade MPG, owing to the >12% of occult intraparotid metastases found with their study.^60^ Similarly, Geiger et al supported the consideration of total parotidectomy for any high‐grade or stage T3‐T4 MPG due to the intraparotid nodal metastases risk.^3^ Meanwhile, Lee et al found that for localized parotid ACC, total parotidectomy did not improve survival compared to partial parotidectomy, supporting the use of a less invasive approach to limit morbidity.^61^

Shabani et al found that surgery alone was an appropriate treatment strategy for patients with less aggressive (grade, neural invasion, tumor size, margins, nodal metastases) salivary gland malignancies.^62^ Partial superficial parotidectomy is also supported for superficial T1 or T2 low‐grade salivary gland malignancies by the ASCO guidelines. Using the National Cancer Database (NCDB), Xiao et al found a low rate of metastatic spread in low‐grade malignancies.^3^, ^63^ Moreover, a study by Zenga et al found a 100% locoregional control rate after a mean follow‐up of 74 months in low‐ to intermediate‐grade MEC that were treated with surgery alone and had narrow surgical margins (≤2 mm).^64^

Moori et al showed, in a meta‐analysis of 5 articles including both benign and malignant parotid tumors, that endoscopic parotid gland surgery can be an effective alternative to conventional approaches with comparable operating times and complications, shorter inpatient length of stay, and improved cosmesis.^65^ In addition, a study by Park et al supported the use of robotic surgery for MPG using a retroauricular incision with facial nerve preservation and reported greater patient cosmetic satisfaction post‐operatively.^66^

#### Management of Lymph Nodes

In MPG, nodal metastasis distribution is dependent on histology and grade. High‐grade cancers, advanced‐stage cancers, and specific histologies, including SDCs and adenocarcinoma, may have a higher incidence of lymph node metastasis.^67^, ^68^ Denis et al demonstrated that the number of metastatic lymph nodes was strongly associated with survival and treatment outcomes of surgically treated patients with high‐grade MPG, and the presence of positive parotid lymph nodes was associated with locoregional treatment failure.^69^ Occult lymph node metastasis rates have been reported at 22% to 32% in clinically N0 (cN0) MPG, which has encouraged investigation on the management of patients with cN0 MPG.^59^, ^70^, ^71^, ^72^, ^73^ French Network of Rare Head and Neck Tumors (REFCOR) recommendations indicate that elective neck dissection (END) depends primarily on the grade of the salivary gland cancer.^74^ Similarly, multiple studies have recommended that patients with high‐grade or advanced‐stage MPG should have an END, including levels II to III, with the inclusion of level IV based on clinical judgment.^72^, ^75^, ^76^, ^77^ Studies have found no OS benefit with END in intermediate‐grade MEC or clinically T1‐2 N0 MEC.^78^, ^79^ The ASCO guidelines recommend elective neck treatment for cN0 patients with high‐grade or cT3‐T4 salivary gland malignancies at presentation.^3^ However, Harbison et al demonstrated that END did not provide a statistically significant effect on survival for patients with high‐grade parotid cancer when taking adjuvant therapy into account, adding controversy to this topic.^80^ Further debate exists for the management of the cN0 neck when FNA or imaging is indeterminate, a limitation that exists with the diagnostic tools to date. In these challenging situations, the literature has supported END or elective neck irradiation, depending on the intraoperative findings of high‐grade disease, perineural or angiolymphatic invasion, and clinical judgment.

### Radiation Therapy

#### Advantages and Disadvantages

Adjuvant radiotherapy has been established as an effective option for MPG.^70^, ^73^, ^81^, ^82^, ^83^ Regarding timing, a delay in the initiation of adjuvant radiotherapy bodes a poor prognosis for patients with major SGCs. Moreover, Patel et al used the NCDB database and found that patients who had indications for adjuvant radiotherapy but did not receive it (missed adjuvant RT) had significantly worse OS (hazard ratio [HR] 1.61, *P* < .001).^84^ Yan et al suggested that radiotherapy should be delivered within 8.5 weeks following the operation, especially for patients with other poor prognostic factors.^85^ Studies show that patients with salivary gland cancer who had adjuvant radiotherapy at the same facility as their surgery were associated with improved OS, supporting the care of MPG at multi‐disciplinary institutions.^84^, ^86^, ^87^

North et al demonstrated that adjuvant radiotherapy independently improved OS (HR 0.34, 95% CI 0.13‐0.88) in patients with intermediate‐grade parotid carcinoma and post‐operative positive margins.^88^ However, in patients with low‐stage and/or low‐grade MPG, surgery alone resulted in a good local control rate regardless of the extent of surgery, without the need for adjuvant RT.^89^, ^90^ Qiu et al investigated the utility of adjuvant radiotherapy in SCC of the parotid using the SEER database and stratifying patients based on age, tumor stage, and nodal involvement. They found that adjuvant radiotherapy significantly improved the OS of patients with intermediate‐risk (older age or advanced tumor stage, 47.5% vs 20.6%, *P* < .001).^91^ Similarly, Hu et al looked specifically at SCC of the parotid and found that adjuvant radiotherapy improved loco‐regional control rates.^92^ In the cN0 patient at the time of treatment, Park et al also recommended adjuvant radiotherapy for patients with high‐grade histology after showing that it led to improved disease control (5‐year OS 88.0% vs 68.8%) in a multi‐institutional study.^93^

#### Proton Therapy

Adjuvant intensity‐modulated radiotherapy (IMRT) has been shown to improve survival for SGC patients, with acceptable toxicities.^94^ However, the use of intensity‐modulated proton beam radiation therapy (IMPT) can offer lower doses to organs at risk (OAR) compared to traditional radiation methods (IMRT, Volumetric Modulated Arc Therapy [VMAT]), leading to the potential for reduced radiation‐related toxicities.^95^, ^96^, ^97^, ^98^ Katagiri et al report that the use of IMPT for MPG has been on the rise in Japan since its approval for head and neck cancers in 2018.^95^ Studies found that IMPT is an effective, safe, and well‐tolerated treatment method for MPG.^99^, ^100^ Future studies comparing IMRT and IMPT for MPG in randomized trials are needed to further delineate the role of IMPT.

### Chemotherapy

The role of chemotherapy in the management of MPGs in the curative‐intent setting remains controversial, particularly when combined with radiotherapy.^101^, ^102^ Numerous studies have shown that the addition of chemotherapy to radiotherapy may increase toxicity without providing significant survival benefits.^103^, ^104^, ^105^, ^106^, ^107^, ^108^ Sousa et al investigated the role of chemotherapy, among other systemic therapies, for SDC and ANOS in a palliative setting and found that the addition of concurrent chemotherapy to adjuvant radiotherapy was not associated with improved OS or RFS.^109^ Chemotherapy can still be considered in patients with advanced, recurrent, or metastatic disease, but its efficacy remains limited. Shen et al utilized the SEER database to demonstrate that adjuvant chemoradiotherapy (CRT) can enhance outcomes in select patients with stage T3/T4 tumors, high‐grade malignancies, or more than five metastatic lymph nodes, compared to adjuvant radiotherapy alone.^110^

When chemotherapy is considered, platinum‐based chemotherapy regimens are commonly used as first‐line treatments for recurrent or metastatic (R/M) salivary gland carcinomas.^101^, ^111^ These regimens have demonstrated efficacy, particularly in high‐grade tumors such as SDC, and are generally well tolerated. Overall, chemotherapy is not currently recommended in the primary treatment of MPG but can be considered in the R/M setting for select patients.

### Immunotherapy

Immunotherapy, namely immune checkpoint inhibitors (ICI), provides advanced oncologic treatment for many cancers, although their role in treating MPG is limited. Existing clinical trials on immunotherapy for malignancies of the salivary gland have been for R/M disease and non‐specific for the primary salivary gland site (see Table 1).^112^, ^113^, ^114^, ^115^, ^116^, ^117^ Ferrarotto et al reported data from a Phase II trial of VEGFR inhibitor axitinib and PD‐L1 inhibitor avelumab in 28 patients with R/M ACC and found an ORR of 18% (5 patients with partial response [PR]) and median progression‐free survival (PFS) of 7.3 months.^118^ Another Phase II trial presented by Vos et al evaluated dual immunotherapy with nivolumab and ipilimumab in patients with R/M salivary gland cancer (32 ACC, 32 non‐ACC) and demonstrated that this treatment had a lower ORR (6%) for R/M ACC compared to other salivary gland cancers (16%), suggesting reduced sensitivity to immunotherapy in ACC R/M salivary gland cancers.^119^ Even et al reported on pembrolizumab outcomes in a Phase II trial of 109 previously treated patients with advanced salivary gland carcinoma and reported PD‐L1 positivity (Combined Positivity Score [CPS] ≥1) of 25.7%.^120^ ORRs were 10.7% versus 2.6% in PD‐L1 positive and negative patients (*P* = .14), respectively.

**Table 1:** Immunotherapy in Malignancies of the Salivary Gland

Study	Year	Immunotherapy	Sites	Patients	Outcomes and findings
Bugia et al	2024	Nivolumab	Parotid gland	1 Metastatic SDC with negative PD‐L1 expression	Partial response
Zhao et al	2023	Sintilimab and CRT	Parotid gland	1 metastatic melanoma to the parotid with complete surgical resection and adjuvant immunotherapy and CRT	Progression‐free survival for more than 19 months
Ferrarotto et al	2023	Avelumab (PD‐L1 inhibitor) and Axitinib	Salivary glands	Phase II clinical trial	ORR: 18%, five patients with partial response
				28 R/M ACC with disease progression within 6 months of enrollment	Median PFS: 7.3 months
					Median OS: 16.6 months
					TRAEs: 29%, all grade 3
Vos et al	2023	Nivolumab and Ipilimumab	Salivary glands	Phase II trial	ACC ORR: 6%
				R/M Salivary gland carcinoma	Non‐ACC ORR: 16%
				64 patients: 32 ACC and 32 non‐ACC	ACC Median PFS: 4.4 months
					Non‐ACC Median PFS: 2.2 months
					ACC Median OS: 30.0 months
					Non‐ACC Median OS: 10.4 months
					TRAEs grade 3 or greater in 38% of patients
Sato et al	2022	Nivolumab or Pembrolizumab	9 parotid gland, 3 submandibular gland	12 salivary gland carcinoma	ORR: 33.3%, 4 achieved complete or partial response
					No PD‐L1 expression analyzed, however, aggressive histologic types were associated with favorable responses
Even et al	2022	Pembrolizumab (200 mg every 3 weeks for up to 35 cycles)	Salivary glands	Phase II KEYNOTE‐158	PD‐L1 expression: 25.7%
				109 previously treated advanced salivary gland carcinoma	ORR: 4.6% (1 complete response, 4 partial response)
					PD‐L1 positive ORR: 10.7%
					PD‐L1 negative ORR: 2.6%
					All responders had responses lasting ≥ 24 months
					Median PFS: 4.0 months
					Median OS: 21.1 months
					TRAE (Grade 3 or 4): 15.6%
Hashimoto et al	2022	Nivolumab or Pembrolizumab	Salivary glands	36 R/M Salivary Gland Carcinoma	Median follow‐up: 6.8 months
					ORR: 11.1%
					All patients who achieved a complete or partial response had positive PD‐L1 expression
Farhat et al	2022	Pembrolizumab	Parotid gland	1 high‐grade MEC	Partial response, allowed successful surgical resection without the need for facial nerve sacrifice
Niwa et al	2020	Nivolumab 240 mg every 2 weeks	Salivary glands	24 R/M salivary gland carcinoma	ORR: 4.2%
					Median PFS: 1.6 months
					Median OS: 10.7 months
					One patient at 28 months without disease progression
					One patient grade 4 elevation in CPK and grade 3 myositis
Lorenz et al	2019	Pembrolizumab	Parotid gland	1 metastatic CASTLE tumor of the parotid initially treated with surgery and radiotherapy with high PD‐L1 expression (>95%)	Partial response
Dalgleish et al	2019	Pembrolizumab and Bicalutamide	Parotid gland	1 AR‐positive SDC	Partial response

Abbreviations: ACC, adenoid cystic carcinoma; AR, androgen receptor; CASTLE, carcinoma showing thymus‐like elements; CPK, creatine phosphokinase; CRT, chemoradiotherapy; MEC, mucoepidermoid carcinoma; OS, overall survival; ORR, overall response rate; PFS, progression‐free survival; R/M, recurrent or metastatic; SDC, salivary duct carcinoma; TRAE, treatment‐related adverse events.

Additional case reports specific to MPG have demonstrated a positive response to immunotherapy for MEC, metastatic melanoma, salivary gland carcinoma, SDC, and carcinoma showing thymus‐like elements (CASTLE) tumors, further highlighting the need for research on which MPG patients would benefit from immunotherapy.^114^, ^115^, ^116^, ^117^, ^121^ Although the efficacy of ICI therapy in R/M SGC is limited, certain patients have been shown to respond and achieve long‐term disease control, highlighting the need for future studies on potential predictive factors for ICI response.

Pathologic biomarkers, including PD‐L1 expression and tumor mutational burden (TMB), have been shown to predict response to ICI therapy in other diseases, however, this remains unclear for MPG.^122^, ^123^, ^124^, ^125^, ^126^ Witte et al investigated rates of PD‐L1 expression (Tumor Proportion Score [TPS], CPS, or Immune Cell Infiltrate Score) in 94 malignant salivary gland tumors and found that only a minority demonstrated detectable PD‐L1 expression.^127^ Similarly, Vital et al analyzed 167 salivary gland carcinomas (118 parotid, 70.6%) and found PD‐L1 expression in 17% of tumors and in 20% of tumor‐infiltrating immune cells (TIIC). The Phase II trial by Vos et al highlights that ACC has a low TMB compared to other salivary gland cancers, consistent with its low response rate.^119^ Finally, Lee et al looked at patients with R/M salivary gland cancer being treated with pembrolizumab and found that patients with a high pretreatment NLR (NLR ≥ 5) were statistically more likely to have early disease progression (at 6 months, HR 12.85, *P* = .005) and death (at 2 years, HR 11.25, *P* = .013).^128^ Overall, biomarker data remains limited, and further studies are needed to clarify their predictive and prognostic value.

### Targeted Drug Therapy

#### Targeted Therapy

Targeted therapies in MPG treatment are being developed; however, current clinical trials include only a limited number of patients and frequently combine different histologic subtypes, making findings challenging to interpret.^129^, ^130^ Personalized therapies are becoming more established options for advanced, recurrent, or metastatic disease, including those positive for androgen receptor expression, human epidermal growth factor receptor type 2 (HER2) expression, tropomyosin receptor kinase (TRK) fusions, and *NOTCH* mutations (see Table 2).^42^, ^101^, ^131^, ^132^, ^133^, ^134^, ^135^ The SalvGlandDx panel is a next‐generation sequencing panel that provides insights into the special molecular characteristics of salivary gland malignancies and provides the potential to inform these targeted therapeutics.^136^

**Table 2:** Targeted Therapy in Malignancies of the Salivary Gland

Target	Studies	Year	Patients	Patients/treatment	Outcomes and findings
**HER2**	Takahashi et al	2024	Salivary glands	Combined data from two Phase I studies	ORR: 58.8%
				17 with HER2‐expressing salivary gland carcinoma	Median PFS: 20.5 months
				Treated with trastuzumab deruxtecan	
				14 had previous HER2‐targeted therapy treatment	
	Lee et al	2023	Salivary glands	Phase II	ORR: 69.8% (30 patients achieved PR, 10 stable disease)
				43 HER2‐positive advanced SDC	Median PFS: 7.9 months
				Docetaxel‐PM and trastuzumab‐pkrb	Median OS: 23.3 months
					Patients with HER2 IHC score of 3+ or HER2/CEP17 ratio ≥2.0 had better efficacy outcomes compared to HER2 IHC score of 2+
	Asper et al	2023	Parotid gland	1 HER2 amplification and AR overexpression metastatic SDC treated with androgen deprivation followed by ado‐trastuzumab emtansine and neratinib	Stable disease for 18 months
	Dahmne et al	2022	Parotid gland	1 HER2‐positive SDC with brain metastasis treated with ado‐trastuzumab emtansine	Partial response in extracranial disease
					Stable brain metastasis
					6‐month follow‐up with stable disease
	Shukla et al	2021	Parotid gland	Metastatic parotid carcinoma with HER2 amplification that progressed after surgery, CRT, neratinib, and ado‐trastuzumab emtansine that was being treated with fam‐trastuzumab deruxtecan	Complete clinical response
					Sustained for at least 7 months
					No adverse events
	Hanna et al	2020	Salivary glands	9 HER2‐positive salivary gland cancer that received adjuvant CRT and trastuzumab	22% (2/9) experienced recurrence
					Associated with improved OS: 74 months with trastuzumab vs 43 months without (*P* = .02)
	Longo et al	2020	Parotid gland	1 HER2‐positive metastatic SDC treated with 6 cycles of trastuzumab and pertuzumab and docetaxel	Partial response
					No TRAE
					18 months follow‐up with stable disease
	Kurzrock et al	2019	Salivary glands	15 HER2‐positive advanced salivary gland carcinoma treated with pertuzumab and trastuzumab	ORR: 60% (1 CR, 8 PR)
					Median PFS: 8.6 months
					Median OS: 20.4 months
	Limaye et al	2013	Parotid gland	5 HER2‐positive parotid primary SDC with metastasis	ORR: 60% (1 CR, 2 PR)
	Krishnamurthy et al	2013	Parotid gland	1 HER2‐positive SDC with disease progression treated with trastuzumab	Partial response
					9 months follow‐up with stable disease
	Kadowaki et al	2013	Parotid gland	1 HER2‐positive SDC with lung metastasis treated with trastuzumab and paclitaxel	Complete response
					Complete disappearance of metastatic lung lesions after 6 cycles
					9 months follow‐up with CR
**AR**	Gogineni et al	2024	Parotid gland	15 AR‐positive salivary duct carcinoma	Median follow‐up: 5.5 years
				Treated with some combination of surgery, adjuvant radiotherapy, and androgen deprivation therapy	5‐year OS: 87%
					5‐year distant progression rate: 13%
					5‐year local recurrence rate: 6%
	Ho et al	2022	Salivary glands	Phase II	ORR: 4.3% (2 PR)
				46 patients with locally advanced/unresectable or metastatic AR+ salivary gland cancers	Stable disease: 52.2% (24 patients)
				Enzalutamide 160 mg daily	
**TRK**	Le et al	2022	Salivary glands	24 patients with TRK fusion‐positive salivary gland cancers	ORR: 92% (13% CR,79% PR)
				Treated with Larotrectinib 100 mg twice daily	78% reached 24‐month PFS
					64% reached 48‐month OS
					No patients discontinued treatment due to TRAEs
***NOTCH***	Hoff et al	2025	Salivary glands	29 patients with metastatic Notch‐activated ACC	ORR: 17% (all PR)
				10/29 patients with major salivary gland AC	Median PFS: 4.2 months
				Treated with NOTCH inhibitors AL101 (gamma‐secretase inhibitor) and brontictuzumab (antibody targeting NOTCH1)	

Abbreviations: ACC, adenoid cystic carcinoma; AR, androgen receptor; CR, complete response; ORR, overall response rate; OS, overall survival; PFS, progression‐free survival; PR, partial response; SDC, salivary duct carcinoma; TRAE, treatment‐related adverse events.

#### HER2 Targeted Therapy

HER2 amplifications have been most commonly found in SDCs, among malignancies of the salivary glands, with a reported expression rate ranging from 25% to 90%.^137^, ^138^, ^139^ Cavalieri et al reported on the prognostic value of HER2 status in R/M androgen receptor overexpressing salivary gland carcinomas (predominantly SDC and ANOS) and found that HER2 overexpression (IHC 3+ or 2+ with positive in situ hybridization) was a negative prognostic factor for disease recurrence (HR 2.97, *P* = .003) and death (HR 3.22, *P* = .007).^140^ However, the prognostic value of HER2 overexpression remains controversial.^109^, ^141^ Hanna et al found targetable HER2 overexpression (IHC 2+ or 3+) in 27 of 52 patients (52%) with malignancies of the salivary glands evaluated with IHC.^142^ Therapeutic options for patients with HER2 amplifications are evolving. Antibody‐drug conjugates, including Fam‐trastuzumab deruxtecan and ado‐trastuzumab emtansine, appear to be well‐tolerated therapeutic options with strong response rates in salivary gland malignancies.^142^, ^143^, ^144^, ^145^ Importantly, the FDA approved fam‐trastuzumab deruxtecan‐nxki for adult patients with unresectable or metastatic HER2‐positive (IHC 3+) solid tumors.

When including malignancies of any salivary gland, multiple clinical trials have found promising response rates. Lee et al examined 43 patients with HER2‐positive advanced SDC treated with docetaxel‐PM and trastuzumab‐pkrb and found an ORR of 69.8%. Hanna et al found an improved 3‐year DFS (100% vs 48%, *P* = .006) and 3‐year OS (100% vs 70*%, P* = .02) in patients with high‐risk histology salivary gland cancers (n = 9) and strong HER‐2 staining intensity treated with adjuvant trastuzumab compared to non‐Trastuzumab treated patients (n = 26).^142^ Kurzrock et al reported a 60% ORR for HER2‐positive advanced salivary gland malignancies in 15 patients treated with pertuzumab and trastuzumab, with a median PFS of 8.6 months.^142^, ^146^ Limaye et al presented a series of 13 patients with SDC and HER2 expression who were treated with trastuzumab either in the adjuvant setting or palliative setting, which included 5 patients with a parotid primary SDC with metastases and showed an ORR of 60% to palliative treatment with trastuzumab.^139^

The majority of parotid gland‐specific case reports that have shown positive clinical response to Trastuzumab have been for R/M SDC.^147^, ^148^, ^149^, ^150^, ^151^ Kadowaki et al presented a patient with metastatic carcinoma ex pleomorphic adenoma of the PG that was treated with combined chemotherapy and trastuzumab for pulmonary metastatic disease and had a complete response and no evidence of recurrence or progression at 13 months.^152^

#### Other Targeted Therapies

Other targeted therapies, including inhibitors for androgen receptors, TRK fusion, and *NOTCH* have been presented in limited studies in malignancies of the salivary glands at any site (see Table 2). In androgen receptor‐positive salivary gland cancers, Fushimi et al looked at combined androgen blockade with leuprorelin acetate and bicalutamide in 36 patients and found an ORR of 41.7% and median PFS of 8.8 months.^153^ Ho et al reported phase II clinical trial data on 46 patients with locally advanced/unresectable or metastatic androgen receptor‐positive salivary gland cancers at any site treated with enzalutamide (androgen receptor inhibitor) and found an ORR of 15.8% and median PFS of 6.0 months.^154^ Gogineni et al looked at 15 patients with androgen receptor‐positive SDC specifically of the parotid and found variable expression of other markers, including HER2.^147^ This supports the development of personalized treatment based on individual histologic profiles for other markers in addition to androgen receptor status.

TRK inhibitors have also been reported for specific MPG, mostly in secretory carcinoma, but can be rarely seen in other histologies.^155^ In a study by Le et al larotrectinib was used to treat TRK fusion‐positive salivary gland cancers in 24 patients of various histologies and salivary gland sites, but showed an ORR of 92%, 78% reached PFS at 24 months, and no patients were discontinued from treatment due to adverse events.^156^ More specifically, Le et al presented a case report of a parotid‐primary low to intermediate grade secretory carcinoma that could not be completely resected because of facial nerve involvement but demonstrated a complete metabolic response following larotrectinib treatment. Finally, Hoff et al looked at *NOTCH* targeting in 29 patients with R/M ACC (82% with *NOTCH* activating mutation) at any salivary gland site and demonstrated a significantly longer PFS with a NOTCH inhibitor treatment compared to previous systemic treatments (HR 0.38 [95% CI 0.19‐0.78], *P* = .0065) and a higher ORR compared to other systemic therapies (17% vs 0%).^135^

### Limitations

This state‐of‐the‐art review is not without limitations. Although this study provides a summary of recent advancements in the management of parotid malignancies, it was done as a non‐systematic review. The use of only PubMed introduces publication bias. Studies often group distinct histologies and grades with differing biologic behaviors, which limits the interpretability of their data. The lack of consistently reported outcomes data and study heterogeneity limits the potential for combined analyses.

### Implications for Practice

Recent advancements in MPG have led to significant changes in the clinical management of these challenging, heterogeneous cancers. The management of the cN0 neck remains controversial, however, multiple studies have supported the inclusion of END in the management of PGMs that are high‐grade or advanced‐stage. Adjuvant radiotherapy may improve patient outcomes, especially in high‐grade cancers. Advances in systemic therapies have led to more personalized treatment of MPG. There is an increased interest in understanding MPG molecular alterations and investigating targeted therapies, such as TRK inhibitors, for MPG with known alterations. Targeted HER2 and AR treatment has already shown promise in clinical practice, warranting more extensive studies.

## Author Contributions

**Anthony Tang**: study design, data acquisition and analysis, interpretation of data, drafting and critical revisions, final approval of version to be published; **Vanessa Helou**: interpretation of data, drafting and critical revisions, final approval of version to be published; **Shaum S. Sridharan**: interpretation of data, drafting and critical revisions, final approval of version to be published; **Renata Ferrarotto**: interpretation of data, drafting and critical revisions, final approval of version to be published; **Jessica L. Geiger**: interpretation of data, drafting and critical revisions, final approval of version to be published; **Dan P. Zandberg**: interpretation of data, drafting and critical revisions, final approval of version to be published; **Diana Bell**: interpretation of data, drafting and critical revisions, final approval of version to be published; **Heath Skinner**: interpretation of data, drafting and critical revisions, final approval of version to be published; **Matthew E. Spector**: interpretation of data, drafting and critical revisions, final approval of version to be published; **Sagar Kansara**: interpretation of data, drafting and critical revisions, final approval of version to be published; **Eugene N. Myers**: study design, interpretation of data, drafting and critical revisions, final approval of version to be published; **Kevin J. Contrera**: study design, data acquisition and analysis, interpretation of data, drafting and critical revisions, final approval of version to be published.

## Disclosures

### Competing interests

Renata Ferrarotto reports personal fees from Regeneron, Sanofi, Merck Serono, Elevar Therapeutics, Prelude Therapeutics, Eisai Inc., Remix Therapeutics, Coherus BioSciences, and Bicara Tehrapeutics and nonfinancial support from Ayala Pharmaceuticals, EMD Serono, ISA, Genentech/Roche, Merck Serono, Pfizer, Viracta, and Gilead outside the submitted work. Jessica Geiger reports personal fees from Merck and consulting fees from Astellas and Aveo Oncology, outside the submitted work. Dr Zandberg reported personal fees from Blueprint Medicines, Macrogenics, Prelude Therapeutics, Merck, Inhibrx, Bicara, Seagen, and Coherus and grants from Merck and BMS to their institution for investigator‐initiated trials outside the submitted work. The other authors declare no conflicts of interest.

### Funding source

None.
